# Circulating differentiated and senescent lymphocyte subsets and incident diabetes risk in older adults: The Cardiovascular Health Study

**DOI:** 10.1002/edm2.384

**Published:** 2022-11-05

**Authors:** Nels C. Olson, Margaret F. Doyle, Petra Buzkova, Sally A. Huber, Ian H. de Boer, Colleen M. Sitlani, Russell P. Tracy, Bruce M. Psaty, Kenneth J. Mukamal, Joseph A. Delaney

**Affiliations:** ^1^ Department of Pathology and Laboratory Medicine, Larner College of Medicine University of Vermont Burlington Vermont USA; ^2^ Department of Biostatistics University of Washington School of Public Health Seattle Washington USA; ^3^ Division of Nephrology, Department of Medicine University of Washington Seattle Washington USA; ^4^ Kidney Research Institute University of Washington Seattle Washington USA; ^5^ Cardiovascular Health Research Unit, Department of Medicine University of Washington Seattle Washington USA; ^6^ Department of Biochemistry, Larner College of Medicine University of Vermont Burlington Vermont USA; ^7^ Departments of Epidemiology, and Health Systems and Population Health University of Washington Seattle Washington USA; ^8^ Department of Medicine Beth Israel Deaconess Medical Center Boston Massachusetts USA; ^9^ College of Pharmacy University of Manitoba Winnipeg Manitoba Canada

**Keywords:** cellular senescence, diabetes mellitus, epidemiology, lymphocyte subsets

## Abstract

**Introduction:**

Cellular senescence is a feature of aging implicated in the pathophysiology of diabetes mellitus (DM). Whether senescent lymphocytes are associated with the future occurrence of DM is uncertain.

**Methods:**

We used cryopreserved peripheral blood mononuclear cells collected from 1860 Cardiovascular Health Study participants (average age 80.2 years) and flow cytometry immunophenotyping to evaluate the longitudinal relationships of naive (CD45RA^+^), memory (CD45RO^+^), senescent (CD28^−^), and T effector memory RA^+^ (TEMRA) (CD28^−^CD57^+^CD45RA^+^) CD4^+^ and CD8^+^ T cells, and memory B cells (CD19^+^CD27^+^), with the risk of incident DM. In exploratory analyses we evaluated the relationships of 13 additional innate lymphocyte and CD4^+^ and CD8^+^ subsets with incident DM risk.

**Results:**

Over a median follow‐up time of 8.9 years, 155 cases of incident DM occurred. In Cox models adjusted for demographic variables (age, sex, race, study site and flow cytometry analytical batch) or diabetes risk factors (demographic variables plus education, body mass index, smoking status, alcohol use, systolic blood pressure, hypertension medication use and physical activity), no significant associations were observed for any CD4^+^, CD8^+^ or CD19^+^ cell phenotypes with incident DM.

**Conclusions:**

These results suggest the frequencies of naive, memory and senescent T cells and memory B cells are not strongly associated with incident DM risk in older adults.

## Graphical abstract

This study performed a longitudinal analysis of a comprehensive panel of 22 innate and adaptive immune cell phenotypes, including differentiated and senescent CD4^+^ and CD8^+^ cells, with incident diabetes risk among a U.S. community‐based population of older adults. The results suggest the frequencies of differentiated and senescent CD4^+^ and CD8^+^ lymphocyte subsets in peripheral blood are not strongly associated with the risk of incident diabetes in older adults free of autoimmune disease.

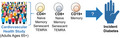

## 1 INTRODUCTION

Age is a main risk factor for diabetes mellitus (DM), which affects more than 20% of adults aged 65 and older.^1^ Cellular senescence, characterized by cell cycle arrest and a proinflammatory senescence‐associated secretory phenotype (SASP), occurs with advancing age and is implicated in several age‐related disorders, including DM.^2^ In murine models of obesity, reducing senescent cells by genetic manipulation or senolytic drugs improved glucose tolerance and insulin sensitivity.^3^

T and B cell quantities and functions change with age and chronic stimulation, referred to as immunosenescence.^4^ Senescent T cells have reduced proliferation in response to antigenic stimulation, restricted T cell receptor diversity and secrete proinflammatory mediators.^4^ Senescence‐associated changes in the T cell compartment are characterized by loss of naive cells, expansion of memory cells and accumulation of progressively differentiated cells, defined by loss of the co‐stimulatory molecule CD28 and re‐expression of CD45RA.^4^, ^5^ Age‐associated changes to the B cell compartment include reduced receptor diversity, increased proinflammatory cytokine secretion and impaired antibody responses.^6^

Through the secretion of proinflammatory SASP products, senescent T cells may contribute to chronic inflammation^4^, ^7^ and promote diabetes risk through direct or indirect mechanisms. Adoptive transfer of senescent CD4^+^ or CD8^+^ T cells induced insulin resistance in murine models,^8^, ^9^ whereas therapies targeting senescent T cells improved glucose homeostasis.^10^ In case–control studies, patients with diabetes had higher blood levels of CD4^+^CD28^−^ and CD4^+^ & CD8^+^ T effector memory RA^+^ (TEMRA) cells compared to those without diabetes.^11^, ^12^ Several cross‐sectional studies have reported an accumulation of CD4^+^ memory cells and loss of naive CD4^+^ cells in those with DM.^13^, ^14^, ^15^, ^16^, ^17^ We recently observed an inverse association of memory B cells with incident type 2 diabetes risk in the Multi‐Ethnic Study of Atherosclerosis (MESA),^18^ consistent with accumulating evidence for a role of B cells in glucose homeostasis and insulin resistance.^19^ The diabetic microenvironment, however, can promote effector cell activation and senescence^20^ and few studies have evaluated cell phenotypes in participants prior to diabetes onset.^18^, ^21^, ^22^ To our knowledge, no studies have investigated longitudinal relationships of differentiated / senescent immune cell subsets with incident diabetes among a cohort of older adults.

We performed immune cell phenotyping using cryopreserved samples collected from participants of the Cardiovascular Health Study (CHS), a U.S. community‐based cohort of adults ages 65 and older, and investigated the longitudinal associations of naive, memory and differentiated/senescent CD4^+^ and CD8^+^ T cell and memory B cell subsets with incident DM over 11.5 years of follow‐up.

## 2 METHODS

### 2.1 Participants

Cardiovascular Health Study recruited 5201 White and African‐American participants in 1989–1990 and 687 primarily African‐American participants in 1992–1993.^23^ Participants were 65 years and older residing in 4 U.S. communities (Forsyth County, NC, Sacramento County, CA, Washington County, MD and Pittsburgh, PA). Semi‐annual follow‐up occurred between 1990 and 1999, alternating between telephone contacts and clinical examinations. Follow‐up by telephone has occurred since 1999. Participants provided written informed consent for participation in the study. Procedures and protocols were approved by Institutional Review Boards.

A series of nested case‐cohort studies was designed in CHS to investigate circulating immune cell subsets as risk factors for cardiovascular diseases (CVD) and related comorbidities. The combined dataset from these studies includes all participants with available cryopreserved peripheral blood mononuclear cell (PBMC) samples collected during the CHS examination in 1998–1999,^24^ which was defined as baseline for this study. By using this combined dataset, the data can be analysed as a simple cohort, with adjustment for analytical batch to account for any confounding induced by the underlying sampling scheme.

### 2.2 Cellular phenotyping

During the 1998–1999 examination, PBMCs were isolated from blood collected into an 8‐ml citrate CPT tube and cryopreserved at −135°C. Cell phenotyping assay methods, reagents and flow cytometry gating strategies are reported in detail.^24^ Samples were thawed at 37°C for 15 min, treated with benzonase and assessed for viability with a live/dead stain. PBMCs were incubated with cell surface markers for 15 min in the dark at room temperature using antibody dilutions recommended by the manufacturer (all antibodies from Miltenyi Biotec).

Naive and memory CD4^+^ & CD8^+^ cells were defined by cell surface expression of CD45RA^+^ and CD45RO^+^, respectively. Memory B cells were characterized by CD19^+^CD27^+^ surface staining. Differentiated/senescent CD4^+^ and CD8^+^ T cells were defined as CD28^−^. TEMRA cells were defined as CD4^+^(or CD8^+^)CD28^−^CD57^+^CD45RA^+^. Additional cell surface labelling assays phenotyped γδ T, natural killer, and regulatory and helper CD4^+^ T cell subsets. The markers used to characterize the immune cell phenotypes are presented in Table S1.

Phenotyping was performed by flow cytometry using a MACSQuant 10 analyser and MACS Quantify software (Miltenyi Biotec). Calibration was performed daily with calibration beads. Compensation was set for each analysis using single colour compensation controls. Negative gates were set using isotype‐matched antibodies with gating strategies previously published.^24^ Cell phenotypes were expressed as proportions of their parent populations (e.g. as percentages of CD4^+^, CD8^+^ or CD19^+^ cells).

### 2.3 Ascertainment and definition of diabetes

Participants with DM at baseline were identified by glucose measurements from blood samples collected in 1992–1993, 1994–1995, 1996–1997 and 1998–1999 and by annual medication inventory assessed through 1999.^25^, ^26^ DM was defined by a fasting (≥8 h) glucose ≥126 mg/dl, random (<8 h) glucose ≥200 mg/dl, or use of oral hypoglycaemic medication. Participants identified with DM between the 1992–1993 and 1998–1999 examinations formed our definition of prevalent DM in this study.

Incident DM was defined as new‐onset diabetes after 1998–1999 through follow‐up until 6/30/2010. Incident DM cases were primarily identified using Centers for Medicare & Medicaid Services (CMS) records which were available for the full CHS cohort. It was defined as ≥2 inpatient, ≥3 outpatient, or ≥1 inpatient and ≥1 outpatient ICD‐9‐CM Medicare claim codes for diabetes over a 2‐year period. During the 1992–1993 CHS examination, this definition had a positive predictive value of 89% for DM as defined by glucose and medication. Incident DM cases were also identified via blood samples collected in a large subset of participants in 2005–2006. Incident insulin use in this study was assessed by participant self‐report via annual telephone calls.

### 2.4 Covariate measurements and definitions

Race was determined by self‐report. Education level was defined by less than high school graduate or high school degree or above. Smoking was defined as never, former (none within past 30 days) or current. Alcohol use was dichotomized as ≤7 drinks per week for women and ≤14 per week for men. Physical activity was assessed as the reported number of “city blocks or the equivalent” walked outside the home in the previous week.^27^ Cytomegalovirus (CMV) IgG antibodies were measured in plasma samples by enzyme immunoassay (Diamedix Corp., Miami Lakes, FL) (coefficients of variation 5.1%–6.8%).

### 2.5 Statistical analysis

Participants with data for at least one immune cell phenotype and ascertainment of diabetes status were included in the analysis (*n* = 1821). Not all cell subsets were measurable in all CHS participants included in the study. Participants with missing data for an individual cell phenotype were excluded from the analyses of that respective subset. The number of participants with data for each of the immune cell assays is presented in Table S1. Variation in the numbers of cell phenotyping assays among participants was due to poor sample quality and technical errors which occurred at random.

Participants with prevalent DM (*n* = 374) or those missing diabetes information during follow‐up (*n* = 24) were excluded from the incident event analyses. Cox proportional hazards models were used to evaluate associations of immune cell subsets with incident DM. We hypothesized a priori that higher circulating proportions of memory (CD45RO^+^), senescent (CD28^−^) and TEMRA (CD28^−^CD57^+^CD45RA^+^) CD4^+^ and CD8^+^ cells and lower proportions of naive (CD45RA^+^) CD4^+^ and CD8^+^ T cells and memory B cells (CD19^+^CD27^+^) would be associated with increased DM risk. In exploratory analyses, we evaluated associations of 13 additional cell phenotypes with incident DM. We also evaluated relationships with incident insulin usage as an exploratory outcome of interest.

Cell phenotypes were modelled singly, per 1‐standard deviation (SD) higher value. Models were adjusted for age, sex, race, CHS clinical site and cell phenotyping analytical batch. A second model adjusted for these variables plus education, BMI, smoking status, alcohol use, hypertension medication use and physical activity.

In secondary analyses, we assessed the cross‐sectional associations of cell phenotypes specified in a priori hypotheses with prevalent DM. Relative risk (RR) models were fit using Poisson regression with adjustments as above.

Sensitivity analyses included adjustment for CMV titres in risk factor‐adjusted models, which are associated with T cell senescence.^28^, ^29^ Bonferroni correction was selected a priori to account for multiple hypothesis testing. The significance threshold was defined as *p* ≤ .0055 for the 9 cell subsets specified in a priori hypotheses. In exploratory analyses evaluating additional subsets, the significance threshold was set at *p* ≤ .0022 (22 total tests).

## 3 RESULTS

The cohort was 83% White, 63% women, with a mean age of 80.2 years and mean (SD) BMI of 27.0 (4.6) kg/m^2^ (Table 1). Among 1423 participants without DM at baseline, we identified 155 incident cases of DM during a median follow‐up period of 8.9 years (interquartile range: 4.7–11.0 years).

**TABLE 1:** Characteristics of CHS participants in 1998–1999 with immune cell phenotyping data (*n* = 1821)

Characteristic	Mean (SD) or *n* (%)
Age	79.6 (4.4)
Male sex, *n* (%)	701 (38.5%)
Black race, *n* (%)	322 (17.7%)
Education (≥high school), *n* (%)	988 (54.3%)
BMI, kg/m^2^	27.0 (4.5)
Systolic blood pressure, mm Hg	135 (21)
Statin use, *n* (%)	289 (15.9%)
Smoking status, *n* (%)	
Never	882 (49.1%)
Former	775 (43.1%)
Current	141 (7.8%)
Alcohol use, *n* (%)	
0	1062 (58.7%)
1–7 per week	564 (31.2%)
>7 per week	183 (10.1%)
CMV, EU/ml (median, 25th, 75th)	144 (62, 246)
Prevalent CHD, *n* (%)	356 (19.5%)
Prevalent heart failure, *n* (%)	66 (3.6%)

BMI, body mass index; CHD, coronary heart disease; CMV, cytomegalovirus.

Table 2 presents associations of the cell populations specified in a priori hypotheses with incident DM, which included memory B cells (CD19^+^CD27^+^) and memory (CD45RO^+^), naive (CD45RA^+^), senescent (CD28^−^) and TEMRA (CD28^−^CD57^+^CD45RA^+^) CD4^+^ and CD8^+^ T cells. In both models, the association of each subset with incident DM was modest and not statistically significant (Table 2). The hazards ratios ranged from 0.92 (95% confidence interval [CI]: 0.77, 1.11) for naive CD4^+^ cells to 1.12 (95% CI: 0.94, 1.33) for TEMRA CD8^+^ cells (all *p*‐values > .05).

**TABLE 2:** Associations of Differentiated and Senescent Lymphocyte Subsets with Incident Diabetes

Cellular phenotype	*n* Diabetes	*N* at risk	Model 1 HR (95% CI)	Model 2 HR (95% CI)
CD4^+^ Subsets				
Naive (CD45RA^+^)	138	1236	0.90 (0.75, 1.07)	0.92 (0.77, 1.11)
Memory (CD45RO^+^)	138	1236	1.11 (0.91, 1.35)	1.09 (0.89, 1.34)
Senescent (CD28^−^)	134	1220	1.09 (0.91, 1.31)	1.06 (0.87, 1.28)
TEMRA (CD28^−^CD57^+^CD45RA^+^)	134	1219	1.03 (0.86, 1.23)	1.03 (0.86, 1.23)
CD8^+^ Subsets				
Naive (CD45RA^+^)	141	1252	1.06 (0.89, 1.27)	1.07 (0.89, 1.28)
Memory (CD45RO^+^)	138	1245	1.00 (0.84, 1.20)	1.01 (0.84, 1.22)
Senescent (CD28^−^)	141	1252	1.01 (0.85, 1.22)	1.01 (0.84, 1.21)
TEMRA (CD28^−^CD57^+^CD45RA^+^)	135	1226	1.10 (0.93, 1.31)	1.12 (0.94, 1.33)
CD19^+^ Subsets				
Memory (CD27^+^)	126	1145	1.00 (0.78, 1.29)	0.95 (0.73, 1.23)

*Note*: Data are hazards ratios (HR) for incident diabetes per 1‐SD higher proportion of immune cells. CI, confidence interval; TEMRA, T effector memory RA^+^.

Model 1: adjusted for age sex, race, Cardiovascular Health Study (CHS) clinical site and analytical batch.

Model 2: Model 1 plus education, BMI, smoking status, alcohol use, systolic blood pressure, hypertension medication use and physical activity.

In exploratory analyses, we evaluated associations of 13 additional innate and adaptive immune cell subsets, including natural killer, γδ T and T helper cells (Th1, Th2, Th17, Treg). None of the cells' associations with incident DM were statistically significant in models adjusted for demographic variables or diabetes risk factors (all *p*‐values >.05; Table 2). When we repeated the analyses using incident insulin usage (*n* = 50), as a proxy for severity of diabetes, as the outcome of interest, we again observed no relationships that were even nominally statistically significant.

Since several studies,^11^, ^12^, ^13^, ^15^, ^16^, ^17^ including our prior work,^14^, ^18^ have reported higher circulating memory, senescent and TEMRA T cells in the blood of those with type 2 diabetes compared with without, we evaluated the cross‐sectional associations of naive, memory, senescent and TEMRA CD4^+^ and CD8^+^ cells with prevalent DM (*n* = 374) at the 1998–1999 CHS examination as a secondary analysis. None of the cells' associations with prevalent DM was statistically significant (all *p* > .05; Table 3). For example, with adjustment for diabetes risk factors, the relative risk estimate was 0.93 (95% CI: 0.84, 1.04; *p* = .16) per 1‐SD higher proportion of CD4^+^ naive cells and 1.09 (95% CI: 0.97, 1.23; *p* = .16) per 1‐SD higher value of CD4^+^ memory cells. In all analyses, interpretation of the results was similar with additional adjustment for CMV.

**TABLE 3:** Cross‐sectional associations of differentiated and senescent lymphocyte subsets with prevalent diabetes at baseline

Cellular phenotype	*n* Diabetes	*N* at risk	Model 1 RR (95% CI)	Model 2 RR (95% CI)
CD4^+^ subsets				
Naive (CD45RA^+^)	309	1592	0.92 (0.83, 1.01)	0.93 (0.84, 1.03)
Memory (CD45RO^+^)	309	1592	1.08 (0.97, 1.21)	1.09 (0.97, 1.23)
Senescent (CD28^−^)	335	1576	0.96 (0.86, 1.07)	0.93 (0.83, 1.04)
TEMRA (CD28^−^CD57^+^CD45RA^+^)	335	1575	1.01 (0.91, 1.11)	0.99 (0.89, 1.09)
CD8^+^ subsets				
Naive (CD45RA^+^)	338	1610	0.96 (0.87, 1.06)	0.94 (0.84, 1.04)
Memory (CD45RO^+^)	338	1603	1.00 (0.91, 1.11)	1.03 (0.92, 1.14)
Senescent (CD28^−^)	338	1610	1.07 (0.98, 1.18)	1.03 (0.93, 1.14)
TEMRA (CD28^−^CD57^+^CD45RA^+^)	333	1577	1.06 (0.96, 1.17)	1.03 (0.93, 1.14)
CD19^+^ subsets				
Memory (CD27^+^)	309	1472	1.00 (0.87, 1.15)	0.99 (0.85, 1.14)

*Note*: Models used Poisson regression.

Model 1: adjusted for age, sex, race, Cardiovascular Health Study (CHS) clinical site, and cell phenotyping analytical batch.

Model 2: Model 1 plus education, BMI, smoking status, alcohol use, systolic blood pressure, hypertension medication use, and physical activity.

Abbreviations: CI, confidence interval; RR, relative risk; TEMRA, T effector memory RA^+^.

## 4 DISCUSSION

Cellular senescence is a widely recognized feature of aging and implicated in DM.^2^ Several cross‐sectional studies,^12^, ^13^, ^15^, ^16^, ^17^ including our prior work MESA,^14^, ^18^ have observed an expansion of memory and contraction of naive CD4^+^ cell proportions in the peripheral blood of those with DM compared to without. Cross‐sectional associations of higher CD28^−^ and TEMRA CD4^+^ and CD8^+^ cells with prevalent diabetes have also been reported.^11^, ^12^, ^22^

Few studies have investigated relationships of immune cell frequencies with the risk of incident DM. We previously evaluated longitudinal associations of T and B cell subsets with incident DM in MESA.^18^ No T cell subsets, including naive, memory, CD28^−^, and TEMRA CD4^+^ or CD8^+^ cells, were associated with DM in this study; however, memory B cells (characterized as CD19^+^CD27^+^) were inversely associated with incident DM risk.^18^ Recent results from the Veterans Aging Cohort Study (VACS) similarly reported T cell subsets were not associated with incident diabetes risk in HIV‐negative participants, whereas higher baseline CD4^+^CD28^−^ and CD4^+^ TEMRA cells were associated with increased diabetes risk in persons with HIV.^21^ Among a Korean cohort at high risk for CVD, CD8^+^CD57^+^ cells were associated with incident hyperglycaemia.^22^

In the present study conducted among CHS participants with a mean age of 80‐years and free of overt autoimmune disease, no T or B cell subsets, including, naive, memory and differentiated/senescent phenotypes, were associated with incident DM risk. These results are consistent with those from MESA and the VACS which included adults with an average age 20–30 years younger than in CHS.^18^, ^21^ The current results are also consistent with null associations of CD8^+^CD28^−^ cells with incident hyperglycaemia among a Korean cohort with a mean age of 64‐years.^22^ In contrast to prior studies, memory and naive CD4^+^ T cells were not associated with prevalent DM in CHS which was unexpected. Null associations with prevalent DM may be explained by differences in the diabetic milieu in older adults, such as, for example, adiposity, postulated to drive T cell activation and senescence,^30^ or by a diminished capacity of T cells to mount a response to DM or its associated features with older age.

Lack of observed associations between differentiated/senescent T cell subsets, and memory B cells, with incident diabetes in CHS may suggest differentiated/senescent lymphocytes play limited roles in the initiation or early progression of DM. Null results may also suggest the importance of other risk factors in promoting diabetes in older age.^31^ The current results, however, do not negate the potential role of differentiated/senescent lymphocytes in exacerbating existing disease among those at younger age or with autoimmune disease. Cells were measured in CHS at a single time point prior to incident DM and it is possible that differentiated/senescent T cells, or their changes over time, are more closely related to acute DM onset, as suggested in one study.^22^

Importantly, we analysed immune cell proportions and not function. It is hypothesized that senescent T cells promote diabetes risk through secretion of proinflammatory SASP mediators, such as interleukin‐6, tumour necrosis factor‐α, and reactive oxygen species,^32^ and whether senescent T cell secretory profiles promote diabetes risk remains an open question. The evaluation of cells in peripheral blood may not reflect inflammation mediated by lymphocytes in the visceral adipose tissue, liver or pancreatic islets suggested as important in some murine and human studies.^8^, ^9^, ^33^ Impaired tissue migration of CD4^+^ and CD8^+^ effector memory and TEMRA cells, however, have been reported among diabetes patients suggesting the sequestration of these cells in the peripheral circulation.^12^

Several additional study limitations are also noted. There was a high degree of missing data for some cell phenotypes. The number of incident DM cases was small, and we cannot exclude the possibility of a type II error. We also had limited data to evaluate diabetes subtypes or severity. Cell surface markers, including CCR7 and CD27, used for characterization of additional memory and senescent T cell subsets were not included in this study. The phenotyping panel primarily included T cells and further studies focusing on the role of B cells in cardiometabolic diseases are required. Strengths of the study include the longitudinal design among a cohort of older adults sufficiently aged to investigate senescence‐associated lymphocyte subsets.

In summary, results from this study of a U.S. community‐based population of adults aged 80 years and older suggest the frequencies of naive, memory and senescent T cells and memory B cells are not strongly associated with DM risk in older adults.

## AUTHOR CONTRIBUTIONS

NCO conceived the study, researched data and drafted and revised the manuscript. MFD conceived the methodology, generated data and edited the manuscript. SAH conceived the methodology and reviewed and edited the manuscript. IHdB reviewed and edited the manuscript. CMS curated data, performed statistical analysis and revised the manuscript. PB performed statistical analysis and revised the manuscript. RPT conceived the study and reviewed the manuscript. BMP conceived the study, edited and revised the manuscript. JAD curated data and reviewed the manuscript. KJM generated data, edited and revised the manuscript. NCO takes responsibility for the contents of the article.

## CONFLICT OF INTEREST

BM Psaty serves on the Steering Committee of the Yale Open Data Access Project funded by Johnson & Johnson. No conflicts of interest are reported for the other authors.

## Supporting information


Table S1

## Data Availability

CHS data can be requested from the Collaborative Health Studies Coordinating Center (CHSCC) upon approval of a manuscript proposal. Instructions are available at https://chs‐nhlbi.org/NewInvest. CHS data is also available via BioLINCC (https://chs‐nhlbi.org/CHS_PublicData).
