# Supplementary material for: Circulating differentiated and senescent lymphocyte subsets and incident diabetes risk in older adults: The Cardiovascular Health Study
Source: Endocrinol Diabetes Metab. 2022 Nov 5;6(1):e384. doi: 10.1002/edm2.384 (PMC9836256; doi:10.1002/edm2.384)
Supplement: Supplementary file 1 — Table S1 [file EDM2-6-e384-s001.docx]

**Supplementary Material**

**Associations of differentiated and senescent lymphocyte subsets with incident diabetes risk in older adults: the Cardiovascular Health Study**

Nels C. Olson, Margaret F. Doyle, Petra Buzkova, Sally A. Huber, Ian H. de Boer, Colleen M. Sitlani, Russell P. Tracy, Joseph A. Delaney, Kenneth J. Mukamal, and Bruce M. Psaty

**Supplemental Table 1.** Markers used for immune cell phenotyping

| **Cellular Phenotype** | **Markers** | **N** |
| --- | --- | --- |
| Natural Killer | CD3^-^CD16^+^CD56^+^ | 1471 |
| γδ T | CD3^+^γδTCR^+^ | 1454 |
| CD4^+^ Naive | CD4^+^CD45RA^+^ | 1592 |
| CD4^+^ Memory | CD4^+^CD45RO^+^ | 1592 |
| CD4^+^ Differentiated/Senescent | CD4^+^CD28^-^ | 1576 |
| CD4^+^ Differentiated/Senescent | CD4^+^CD57^+^ | 1591 |
| CD4^+^ Differentiated/Senescent | CD4^+^CD28^-^CD57^+^ | 1576 |
| CD4^+^ TEMRA | CD4^+^CD28^-^CD57^+^CD45RA^+^ | 1575 |
| CD4^+^ Activated or mature | CD4^+^CD38^+^ | 1591 |
| Th1 | CD4^+^CXCR3^+^CCR4^-^CCR6^-^ | 1241 |
| Th2 | CD4^+^CXCR3^-^CCR4^+^CCR6^-^ | 1241 |
| Th17 | CD4^+^CXCR3^-^CCR4^+^CCR6^+^ | 1241 |
| T regulatory | CD4^+^CD25^+^CD127^-^ | 1456 |
| CD4^+^ Activated or T regulatory | CD4^+^CD25^+^ | 1456 |
| CD8^+^ Naive | CD8^+^CD45RA^+^ | 1610 |
| CD8^+^ Memory | CD8^+^CD45RO^+^ | 1603 |
| CD8^+^ Differentiated/Senescent | CD8^+^CD28^-^ | 1610 |
| CD8^+^ Differentiated/Senescent | CD8^+^CD57^+^ | 1452 |
| CD8^+^ Differentiated/Senescent | CD8^+^CD28^-^CD57^+^ | 1610 |
| CD8^+^ TEMRA | CD8^+^CD28^-^CD57^+^CD45RA^+^ | 1577 |
| CD8^+^ Activated or mature | CD8^+^CD38^+^ | 1609 |
| Memory B cell | CD19^+^CD27^+^ | 1472 |

TEMRA, T effector memory RA^+^

**Supplemental Table 2.** Cox proportional hazards models of incident diabetes per 1-SD increment of immune cells included as secondary analyses.

| **Cellular Phenotype** | **n Diabetes** | **N at Risk** | **Model 1**  HR (95% CI) | **Model 2**  HR (95% CI) |
| --- | --- | --- | --- | --- |
| Natural Killer | 126 | 1144 | 1.04 (0.85, 1.27) | 1.07 (0.87, 1.32) |
| γδ T | 123 | 1130 | 1.01 (0.83, 1.23) | 1.03 (0.84, 1.25) |
| CD4^+^CD57^+^ | 138 | 1235 | 1.04 (0.86, 1.25) | 1.02 (0.84, 1.24) |
| CD4^+^CD28^-^CD57^+^ | 134 | 1220 | 1.01 (0.84, 1.22) | 1.01 (0.84, 1.22) |
| CD4^+^CD38^+^ | 140 | 1234 | 0.98 (0.82, 1.16) | 0.99 (0.83, 1.18) |
| Th1  (CD4^+^CXCR3^+^CCR4^-^CCR6^-^) | 115 | 948 | 1.09 (0.91, 1.32) | 1.08 (0.89, 1.31) |
| Th2  (CD4^+^CXCR3^-^CCR4^+^CCR6^-^) | 115 | 948 | 1.17 (0.97, 1.40) | 1.09 (0.89, 1.33) |
| Th17  (CD4^+^CXCR3^-^CCR4^+^CCR6^+^) | 115 | 948 | 1.01 (0.83, 1.23) | 0.98 (0.80, 1.21) |
| T regulatory  (CD4^+^CD25^+^CD127^-^) | 123 | 1130 | 1.05 (0.86, 1.28) | 1.07 (0.87, 1.30) |
| CD4^+^CD25^+^ | 123 | 1130 | 1.08 (0.86, 1.35) | 1.03 (0.81, 1.30) |
| CD8^+^CD57^+^ | 126 | 1120 | 1.15 (0.97, 1.37) | 1.16 (0.97, 1.39) |
| CD8^+^CD28^-^CD57^+^ | 141 | 1252 | 1.08 (0.91, 1.28) | 1.08 (0.91, 1.29) |
| CD8^+^CD38^+^ | 143 | 1250 | 1.03 (0.85, 1.25) | 1.03 (0.84, 1.25) |

CI, confidence interval; HR, hazards ratio.

Model 1: adjusted for age, sex, race, CHS clinical site, and cell phenotyping analytical batch.

Model 2: Model 1 plus education, BMI, smoking status, alcohol use, systolic blood pressure, hypertension medication use, and physical activity.
